# Monitoring Health Parameters of Elders to Support Independent Living and Improve Their Quality of Life

**DOI:** 10.3390/s21020517

**Published:** 2021-01-13

**Authors:** Ilia Adami, Michalis Foukarakis, Stavroula Ntoa, Nikolaos Partarakis, Nikolaos Stefanakis, George Koutras, Themistoklis Kutsuras, Danai Ioannidi, Xenophon Zabulis, Constantine Stephanidis

**Affiliations:** 1 Foundation for Research and Technology Hellas, Institute of Computer Science, N. Plastira 100, Vassilika Vouton, GR-70013 Heraklion, Greece; iadami@ics.forth.gr (I.A.); foukas@ics.forth.gr (M.F.); stant@ics.forth.gr (S.N.); partarak@ics.forth.gr (N.P.); nstefana@ics.forth.gr (N.S.); ioanidi@ics.forth.gr (D.I.); cs@ics.forth.gr (C.S.); 2 Department of Music Technology and Acoustics, Hellenic Mediterranean University, GR-71410 Rethymno, Greece; 3 OPENIT, Idaiou Androu 9, GR-71202 Heraklion, Greece; koutras@openit.gr (G.K.); kutsuras@openit.gr (T.K.); 4 Department of Computer Science, University of Crete, GR-70013 Heraklion, Greece

**Keywords:** mHealth, eHealth, quality of life, elderly, independent living, sensors, monitoring of vital signs, virtual assistant, cough detection, human-centred design

## Abstract

Improving the well-being and quality of life of the elderly population is closely related to assisting them to effectively manage age-related conditions such as chronic illnesses and anxiety, and to maintain their independence and self-sufficiency as much as possible. This paper presents the design, architecture and implementation structure of an adaptive system for monitoring the health and well-being of the elderly. The system was designed following best practices of the Human-Centred Design approach involving representative end-users from the early stages.

## 1. Introduction

According to the World Health Organisation (WHO), the pace of population ageing is much faster than in the past. By 2050, the world’s population aged 60 years and older is expected to total 2 billion, up from 900 million in 2015 [1]. Aging according to Strehler’s criteria is a naturally evolving process that is universal, intrinsic, progressive and deleterious. It affects the adaptability, sensitivity, accuracy and sustainability of the living organism by gradually reducing it [2,3]. Elderly people show great heterogeneity in terms of their characteristics and thus gerontologists have divided them into subgroups as the reduction of normal biological functions becomes greater from one age group to another. In fact, the observed increase in the number of people reaching age 65 globally—coupled with the increase in their life expectancy—has expanded the classification of the elderly to include three sub-populations commonly referred to as the “young-old, (age 65–74)” the “old (age 74–84),” and the “old-old (age 85+)” groups [4]. The increase in the population of elderly people has brought an increase in the demand for medical services, special care, a larger number of hospitalisations and the necessity of creating additional places in institutions for elders. One way to counteract this dynamic is to provide ways to support the maintenance of the quality of life of the elders. The longer people can remain mobile and care for themselves, the lower are the costs for long-term care to families and society.

In general, the effort to maintain the quality of life at a certain level is closely related to coping with the various forms of chronic illnesses, such as cardiovascular diseases, chronic respiratory diseases, diabetes, and mild cognitive impairment, which often concur with ageing [5]. In this respect, mobile health (mHealth) solutions are increasingly being used for chronic disease management, but also for patient communication, monitoring, and education [6]. When it comes to mHealth for older adults, it has been found that it enables a perception of independence, however, there are still challenges emanating from the existing complexity of technology and medical literacy of the target user group [7]. Factors that have been found to influence the acceptance of mHealth solutions by older adults include performance expectancy, effort expectancy, social influence, technology anxiety, and resistance to change [8].

In this context, this paper proposes, the MyHealthWatcher (MHW) system, a health monitoring system, which aims to provide a user-friendly way for elderly people to self-manage their general health and for their healthcare professionals and caregivers to monitor their health status. The proposed system consists of three main modules: a mobile application for the elderly people which can connect to sensors that provide continuous readings of common vital signs (e.g., heart rate, blood oxygen level, and stress); a sound processing subsystem for speech recognition and detection of pathological conditions (e.g., coughing) in acquired sound signals; and, a web-based application for the healthcare professionals and caregivers of the elderly people who are given access rights to the health data.

Through the system’s mobile application, the elderly people are able to view their vital signs captured by the sensors, manually input other vitals readings from devices not included in the system (e.g., blood pressure and blood glucose), give access rights to the collected health data to their chosen healthcare professional or caregiver, and receive personalised messages and guidelines by the latter. The mobile application module also explores the implementation of a virtual assistant (avatar) service, which allows verbal interaction between the application and the elderly user. Specifically, the assistant will be able to communicate verbally and textually any requested information about current vitals and messages received from their connected healthcare professionals. At the same time, the speech recognition module of the sound processing subsystem will allow the users to “ask” the assistant about such information through speech commands.

Lastly, through the web-based application which interconnects with the mobile application, the healthcare professionals or caregivers are able to monitor the overall well-being of their elderly patients, be alerted and notified of detected measurements outside the normal range, and send personalised messages and instructions. In addition to the technological contributions, this work also contributes a comprehensive concept which was developed following a Human-Centered approach, actively involving end-users and stakeholders in the requirements specification, design and evaluation phase.

The remainder of this paper is structured as follows: related work in the field of mHealth solutions for older adults is discussed in Section 2; the methodology followed for the development of myHealthWatcher is presented in Section 3; the context of use and requirements analysis are presented in Section 4 and Section 5 correspondingly; the myHealthWatcher system is presented in Section 6 and its evaluation with end-users in Section 7; finally, a discussion and directions for future work are presented in the concluding Section 8.

## 2. Related Work

Mobile health has been characterised as “the healthcare revolution”, mainly due to its potential to revolutionize healthcare services beyond temporal, geographical and even organisational barriers [9]. This potential becomes of paramount importance when it comes to older adults, not only improving their quality of life but also assisting the healthcare system—effectively and efficiently supporting them. In this respect, numerous efforts have been reported in literature targeted at providing mHealth solutions to the elderly, which have been classified as continuous monitoring, supervised healthcare, assisted healthcare and self-healthcare management [10]. In all cases, mHealth solutions typically involve sensors for acquiring health-related measurements, however, they differ substantially in how health data are managed: in self-healthcare management, the only stakeholder is the data subject (the older adult); in assisted healthcare, caregivers are notified in the case of abnormal measurements; in supervised healthcare, caregivers are more actively involved in health monitoring, regularly receiving information about the patient and not only in the case of abnormal measurements; finally in continuous monitoring solutions, a fully automatic analysis of real-time vital signs of the patient is supported, thus providing automated responses in addition to the remote non-automatic clinical analysis by a specialist (caregiver). This paper reports a solution spanning all four levels, handling health data in a flexible approach, based on the preferences of the older adult and the criticality of their health condition.

The high processing power of contemporary mobile devices along with their potential for interconnectivity with various other devices (e.g., sensors, wearable, or medical devices) has spurred the development of mobile solutions for monitoring various physiological measurements. Such measurements include heart activity, blood pressure monitoring, glucose level analysis, respiratory activity, and body temperature. Each solution combines one or more of the aforementioned measurements, in an ad-hoc manner, principally driven by a specific project or user requirements. For instance, heart activity, blood pressure, and glucose are monitored and managed in [11], glucose levels and heart activity are addressed by [12], pulse and temperature signals are recorded by [13], a pulse oximeter and GPS data are employed by [14], while cardiac, respiratory and motor activity are monitored by [15]. Systematic reviews of mobile health applications provide lists and insights on the various solutions that have been developed [7,9,10,16].

Solutions in the field of mHealth, targeted at older adults, vary with regard to their aims and objectives, including solutions for disease management (such as diabetes control, hypertension control, adherence to medication, depression treatment, and psychological support) as well as solutions for pursuing behavioral change (such as smoking cessation, or healthy diet and lifestyle) [17]. It is notable, that although a plethora of solutions exists in the field of mHealth, only a limited number are targeted to older adults, having actively involved them in a systematic manner in the design and evaluation of the implemented solutions. For example, senSAVE, a mobile health monitoring system, addressing older adults with cardiovascular diseases, measuring oxygen saturation, blood pressure, as well as pulse and pulse wave transit time, was evaluated by 22 older adults with regard to the information presentation, interaction design, and overall usability; however, target users were involved only in the evaluation phase and no requirements analysis results have been reported [18]. eCAALYX is another mobile app targeted at older people with multiple chronic conditions, acquiring health-related information through sensors so as to detect anomalies such as tachycardia or signs of respiratory infections; however, no evaluation results or requirements from the target user population have been defined [19].

Recently, mobile devices have also been used in the mHealth context towards detecting pathologies in patients’ cough, a development mostly stemming from advancements in the quality of acoustic sensors and processing capacity of smartphones [20]. Two main challenges faced in smartphone-based cough detection are noisy input signals, as well as the highly demanding algorithms—in terms of battery consumption—that need to run on the device [21]. Nevertheless, current research efforts report positive results in terms of accuracy [22,23,24], but also in terms of sensitivity and in noisy environments and battery consumption [21]. However, an important remark is that all the cough detection systems mentioned so far are trained and tested on specific acoustic sensing equipment while in many cases, a specific placement of the sensing device with respect to the patient is required [25,26,27]. To our knowledge, the work described in this paper is the first to include, in a unified mobile application, monitoring of vital signals and cough detection, all used in a coordinated manner to provide a comprehensive overview of the chronic patient’s/elderly person’s health status.

Motivated by the need for maintaining elderly users actively involved with assistive mobile applications, and enhancing overall acceptance, several efforts have suggested using virtual agents as assistants. Virtual characters can be well suited as intuitive virtual agents for elderly companions, due to their inherent ability to simulate verbal as well as nonverbal communicative behaviour. This type of interface is made possible with the help of multimodal dialog systems, with additional modalities just like in human–human interaction [28]. However, employing virtual characters as personal and believable dialog partners in multimodal dialogs entails several challenges, because this requires not only a reliable and consistent motion and dialog behaviour but also regarding nonverbal communication and affective components. Besides modelling smart behaviour, which is an active field of research in AI [29], the visual representation of a character including its perceivable behaviour, from a decoding perspective, such as facial expressions and gestures [30], implicates many open issues concerning natural communication [31]. In this direction, recent approaches combine the usage of state of the art motion capture equipment (e.g., [32,33]) and modern multi-platform game engines together with motion segmentation and motion retargeting techniques as a means of transferring natural human-like behaviour to virtual characters [34,35].

A study on user preferences employing focus groups and interviews confirmed the social effects of virtual humanoid agents and highlighted the need for participatory design approaches to enhance the acceptability of the target user group [36]. A study on the voice of humanoid agents highlighted that voice is very important for enhancing elders’ acceptance of virtual humanoid agents [37]. Several other studies can be found in the literature regarding the attributes a humanoid avatar should have in order to be acceptable by older adults, while such agents have been reported to be used in companionship and home care assistance applications [38].

In summary, mHealth technologies have been widely studied and deployed for chronic patients, but also for assisting home health monitoring for older adults. Applying best practices in the field, this work proposes MyHealthWatcher (MHW), a solution for monitoring the vital signs of a person, combined with an innovative cough detection system for identifying potential pathologies and providing a more complete representation of the person’s health status. At the same time, in order to enhance acceptance and assist the elderly in using the application, MHW employs a humanoid avatar, assisting users in carrying out and recording measurements. Last, but not least, the development of the described application has followed a human-centred approach, actively involving end-users (older adults, caregivers, and medical doctors), thus delivering a system that addressed in the best possible way the—sometimes diverging—requirements of all the target user groups.

## 3. Methodology

The design and development of the proposed system followed the best practices of the Human-Centred Design (HCD) approach. HCD is an iterative process for the design and development of interactive applications and systems, which places the targeted users of the system and any other identified stakeholders’ needs at the centre [39]. By focusing on users’ needs and requirements and applying human factors principles and techniques in the design and development phases ensures that the produced system or application will be usable and useful.

By actively involving end-users in the design and development process of the MHW system, we were able to explore the following research questions:

- R1: What are the habits of elderly people with regard to self-monitoring their health at home?
- R2: What would elderly users require from an mHealth solution?
- R3: Are healthcare professionals positive towards employing such mHealth solutions for self-monitoring of health status when it comes to their older patients?

The exploration of the above research questions helped us collect valuable information, which played an integral role in the formation of the user requirements of the system and ultimately in its design and implementation. The collection of the information and the specification of the user-requirements took place in stages 1 and 2 of the HCD process, the design of the system in stage 3, and the evaluations in stage 4. An overview description of these stages is provided below, while a full analysis is provided in the respective sections that follow.

Stage 1: In this stage, we focused our activities on understanding the context of use for our proposed system. This included specifying the potential users of the system, their characteristics, general needs, goals, and motivations. Additionally, it included understanding the environment in which they operate, e.g., physical, technical, social. Methods employed in this stage included literature research on age-related topics, market research on existing systems and devices that monitor health parameters, brainstorming sessions with the project team, and interviews with two occupational therapists with great experience in working with elderly people. The output from this stage was the description of the user groups of the system.

Stage 2: In this stage, we focused on the specification of the requirements that the system will need to meet to satisfy the identified from the previous stage user needs and support their goals. This regards the following: specification of functional and non-functional requirements; description of the system functionality that is important to the users and that satisfies their objectives; description of the characteristics that the system should have in order to adhere to the specifics of each context of use and to common ergonomics and usability standards that are important for proper operation of the system. Methods employed in this stage to collect information from representative target users regarding health-related topics included questionnaires and semi-instructed interviews. Additionally, brainstorming meetings among the members of the project team were conducted to finalise the requirements based on the analysis of the collected information. The output of this stage was a set of use cases and scenarios of use, which described in detail the functionality and tasks that the two applications, the mobile application and the web-based application needed to support, as well as the workflows, or steps, that the user would need to follow to complete supported tasks.

Stage 3: In this stage, we developed the system prototype. This included the design of the architecture of the envisioned system and the two main applications, the mobile application for the elderly people and the web-based monitoring application for their healthcare professionals and caregivers. The initial design prototypes were produced in the form of medium-fidelity static mock-ups by an interaction designer based on the requirements and specifications from the previous two stages. These mock-ups were used by the project team to facilitate the conceptualisation of the end applications and the overall system. Once they matured, they were then evaluated by usability experts and subsequently by representative users from the identified target audience as described in the next stage. The final versions of the UI design prototypes were then used for the actual implementation of the system.

Stage 4: This stage of our process included the evaluations of the designed prototypes from the previous stage by usability and domain experts and by representative users of the target audience. The method employed in the first case was that of heuristics inspection technique [40], a common expert-based evaluation technique used in Human-Computer Interaction (HCI). In this technique, an HCI expert inspects the prototypes and identifies areas or interactive elements that violate usability standards and guidelines and could cause problems to the user. Their judgement is based on prior experiences and knowledge of common human factors and ergonomics guidelines, principles, and standards. Two HCI usability experts participated in these evaluation iterations. The results from their inspections were used to improve the prototypes. The improved prototypes were then evaluated by representative users from the target audience. Specifically, the mobile application prototype was evaluated by 16 elderly people in the context of a participatory evaluation workshop that was conducted. The web-based application prototypes were evaluated by domain experts of the medical field and one in the field of occupational therapy. The experts were shown high-fidelity working prototypes and were asked to perform a series of common tasks and using the Think-Aloud method to express their thinking process, concerns, and opinions. The evaluation provided valuable information that was used to improve the designs prior to their implementation.

Finally, in this stage, separate evaluations were performed in order to assess the performance of the speech recognition and the cough detection components of the sound processing subsystem. The results of this evaluation are also reported in Section 7.3.

All the activities that required the involvement of human subjects, in the user requirements and evaluation phases of the project presented in this paper, have received the approval of the Ethics Committee of the Foundation for Research and Technology—Hellas (Approval date: 1 March 2019/Reference number: 35/1-3-2019). No clinical data were collected during the present study. User consent forms were given to each subject to sign prior to their participation in any of the activities in accordance with the European Union GDPR regulation [41].

## 4. Context of Use

As the goal of the MHW system is to promote and assist in the self-management of typical age-related chronic health issues, the primary user target group is elderly people (age 65+) who either live alone at home, or with another family member and are generally capable of self-managing their health under the guidance of their primary healthcare professional. Their main goal is to continue living an independent life at home, be able to carry out their daily activities, and overall continue to experience a good quality of life. However, usage of the system is not limited to independent living elderly people. It could also be used by people who are semi-independent, such as those living in their own unit under the care of a nursing institution, or those who live in their own home, but receive some sort of daily assistance and care. In fact, according to Eurostat’s statistics on Ageing Europe, across the EU Member States, “informal care services are increasingly being recognised as part of the long-term care system, rather than something that takes place in the isolation of family homes.” [42].

In all these cases, the elderly persons can use the MHW system, the selected sensors and mobile application, to be informed on the status of basic vital signs (e.g., heart rate, blood oxygen, stress, respiratory condition), to input manually health measurements collected by other medical home devices (e.g., blood glucose, blood pressure), and share the data with their personal healthcare professional and/or a caregiver from their close environment. Furthermore, through the system, the elderly person can choose who from their contacts should be notified when any of the sensors capture vital readings that are outside the normal range. In all these scenarios of use, it is important, for ethical and privacy reasons, for the elderly person to make all the decisions regarding what data they want the system to track, who will have access to these data besides themselves, and who will be notified in the case the system captures data that is off the normal range. These decisions will be propagated to the system via the application settings and preferences.

The second group of users of the project concerns healthcare professionals, mainly physicians in medical fields that deal with the management of chronic diseases common to the elderly, such as pathology, special pathology, general medicine, cardiology and pulmonology. The attending physician who works within the primary and secondary health structure, in addition to offering medical services to their elderly patients, also provides advice and guidance for long-term self-management of their health problems. This creates a framework for the long-term follow-up of the elderly by their physician. In this context, the physician would use the web-based application of the MHW system as a monitoring tool which will give access to their patients’ health indicators data collected by the sensors. Through the system, physicians will also be able to choose when and how to be notified of abnormal health values regarding an elderly patient that they want to monitor closely and send personalised messages and guidelines to their patients. In this user group, we included that of non-professional caregivers (e.g., relatives, friends) who often are actively involved in supporting the elderly person in the management of their health. An example of this context of use would be a son or daughter who lives far away from a parent or relative that lives on their own and would like to be able to monitor that person’s overall health status from afar in order to be able to intervene when necessary.

## 5. User Requirements Specification

User requirements describe how the system should behave and function based on the user’s expectations and motivations. The method used for user requirement elicitation was that of questionnaires and semi-structured interviews as mentioned in the methodology section. Specifically, three separate questionnaires were prepared, one for each user group described previously. The questionnaire for the health professionals and informal caregivers had about the same questions with slight differences in the wording. The three questionnaires were prepared by the HCI team of the system and were tested in a pilot run with seven people (three elderly people, two doctors, and two caregivers) to determine the appropriateness of the questions. The questionnaire for the user group “Elderly users” was also reviewed by an occupational therapist, who gave guidelines for writing the questions in a style that would be appropriate for the elderly (e.g., avoid technical jargon, keep questions short, avoid ambiguous statements). Feedback collected during the pilot run was used to improve the questionnaire before the distribution to the actual participants in the study.

For the questionnaires, we used both closed and open-ended questions. The purpose of the closed questions was to gather some quantitative data, while the open questions were used to gather the quality type of data, such as comments, opinions and ideas on how a health monitoring system should function. The sample of the elderly was selected from the general population of 65 and older in Greece, and included people who live alone or with a family (spouse, child, brother, etc.), independently, or in a local nursing home facility. A total of 45 people answered this questionnaire.

The sample of participants in the user group of health professionals was selected with the basic criterion of being healthcare providers that have experience in treating age-related health problems and working with elderly patients, such as doctors, nurses, and occupational therapists. In the sample, we also included social workers who work in a community care centre because of their extensive experience and knowledge of older people’s needs, goals, and expectations. In total, the sample of this group included four physicians, eight nurses, two occupational therapists, one physiotherapist and three social workers. Of the 16, 18 answered through the questionnaire, while two of the doctors answered in the form of a semi-structured interview in order to gather more detailed information about the follow-up needs of the elderly. Finally, a small targeted sample of 10 people was selected from the third group of users, the caregivers with the basic criterion of caring for elderly people in their family environment or people through a community home help program.

The questionnaire for the elderly differed from that of the healthcare professionals and caregivers in that it had some additional questions regarding the current status of the overall health of the elderly, their current techniques for taking health measurements at home, and their use of modern technologies (e.g., smartphones, tablets, computers) and applications (e.g., email, mobile applications).

The results of each questionnaire and the results of the semi-structured interviews are analysed separately in the subsections that follow.

### 5.1. Questionnaire Results—Elderly People

A total of 45 people in this user group answered the questionnaire. The characteristics of the participants are shown in Table 1 and the answers they gave to each question are presented afterward.

Question: “Select from the list below, the technology devices that you use and the number of times you use them. Select all that apply”.

When it comes to the participants’ usage of technology, 11 answered that they use a smartphone or tablet on a daily basis, and 8 answered that they use a computer on a daily basis. That translates to 19 out of 45 people of the sample population saying that they use modern technology on a daily basis Table 2. None of the 16 people living in the nursing home use any of the above technologies. So, if we exclude this sample from the total, it means that from the people living at home 19 out of 28 use modern technologies.

Question: “Select from the list below, which electronic applications you use and the number of times you use them”.

Moreover, 14 of the 28 using modern technologies said that they use applications such as emailing, social media channels, and other mobile apps (e.g., e-banking) on a daily basis and an additional five of them use those on a weekly basis (Table 2). These results exceeded our expectations.

Although the sample used in this study is too small to make any generalised conclusions, it may be an indication of a shift observed in the use of modern technologies by older people [43]. There is an undeniable digital divide between the generations in terms of their access to and use of modern information and communications technologies (ICTs). According to Eurostat statistics, there were still one quarter (25%) of people aged 55–64 years and more than two fifths (44%) of people aged 65–74 years in the EU-27 in 2017 who had never used a computer [42]. However, in the same analysis, their data showed that between 2008 and 2017, the share of the EU-27 population aged 55–64 years never having used a computer fell from 47% to 25% (with a reduction recorded for every Member State) while for the age group 65–74 years the share fell from 70% to 44%. They also state that older people are likely to make far greater use of ICTs in the future, given the continuing digitalisation of society and an increasing number of people with ICT skills passing into older age [42].

Question: “How would you rate your overall health status?”

**Figure 1:**
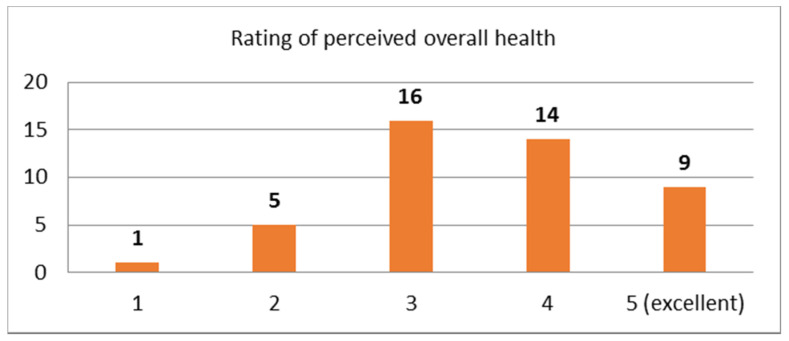
Distribution of rating scores for perceived overall health.

The perceived overall health status received an average score of 3.5 (STDEV 1.0) with the majority of the participants rating their general health from 3 and above in the provided 5-item Likert scale (5 = excellent) as it is depicted in Figure 1.

Question: “Do you suffer from a chronic illness? If you answered yes in the previous question, rate from 1–5, with 5 being very difficult, how difficult is the management of the illness in your daily life?”

**Figure 2:**
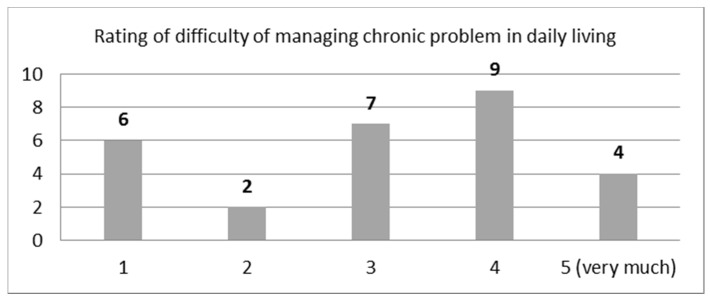
Distribution of rating scores for difficulty in managing chronic illness in daily life (answers from 28 participants who answered that they have a chronic illness).

In the first part of the question “do you suffer from any chronic health problems”, 28 out of 45 said that they do suffer from a chronic illness. Of the 28 who said they have a chronic ailment, 20 of them also said that they experience moderate to high difficulty in managing their chronic problem. The question received an average rating score of 3.1 (STDEV = 1.37). The distribution of the rating scores is depicted in Figure 2.

Question: “Select from the list below the medical devices you use at home. Select all that apply”?

The list of options included a blood pressure meter, a blood glucose meter, a blood oxygen meter, and others, and participants could select more than one option. The results of how many selected each option are shown in Table 3. The answers to this question show the basic types of self-monitoring practiced at home. It is noted that even some people who answered that they do not have a chronic illness, said that they used a medical device at home.

Question: “How do you keep a record of results from the medical devices used at home?”

Excluding the participants that live in a nursing home, 13 out of the 28 people that live independently, do not keep a record of the results of their measurements anywhere (Table 4).

When asked whom they inform about their measurements currently, based on the answers of 43 people, 17 people said just their doctor, 5 said just their relative, 9 said no-one and 12 people selected more than one option (Table 5).

These results show that there is a diverse approach from the selected sample of participants when it comes to whom they want to share their health data with, which means that any monitoring system should provide options for specifying their own preferences.

Hypothetical scenario questions

The following hypothetical scenario was given to this user group: “Imagine an electronic wristwatch which, when you wear it, it automatically measures your heart rate, blood oxygen, body temperature, etc. The results of these measurements are automatically saved in a special program on your mobile or tablet. Based on the above scenario, please answer the following questions.”

Question: “How often would you like for the hypothetical system to notify you about the results of your health measurements?”

Answers to this question were split, with 17 saying one or two times on a daily basis, 17 said only when a result is outside the normal range, and 7 said that they would prefer to check their results on demand (Table 6).

Question: “Besides you, who else would you want to be able to see your health measurement results?”

This question allowed for multiple selections from the following options: My physician, my relative, my home assistant or caregiver, other. Answers to this question, are presented in Table 7. These data show that elderly people are open to the idea of sharing health data with people in their closed environment.

Question: “How would you like for the system to notify the above people?”

The results of this question are shown in Table 8 below. The majority selected the automatic notification method option either for all results or for abnormal results only. Some elderly people selected the option to have control over when and whom to notify.

Question: “From 1–5, with 5 being excellent, how would you rate your quality of life in general?”

**Figure 3:**
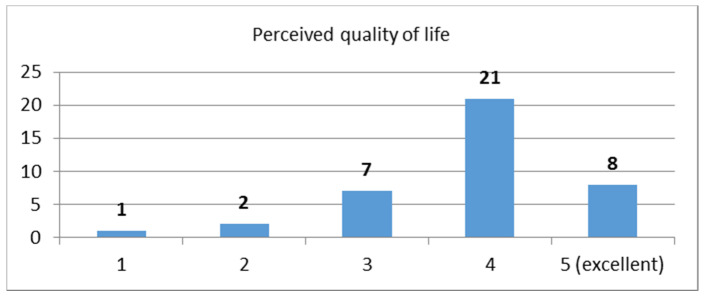
Perceived quality of life ratings.

Lastly, the question about the perceived level of quality of life received an average rating score of 3.72 (STDEV = 0.97). The distribution of the scores is depicted in Figure 3.

In the open-ended support question, “what does quality of life mean to you”, good health and independence/self-sufficiency, were the two that were mentioned the most receiving 17 and 11 mentions respectively.

### 5.2. Questionnaire Results—Healthcare Professionals

Table 9 shows the characteristics of the participants in the healthcare professionals user group.

Hypothetical scenario questions

The following hypothetical scenario was given to this user group: “Imagine that some of the patients you monitor use an electronic system in which they record the results of various vital signs measurements that they take as part of their daily health monitoring. Some of the measurements are taken through common medical devices (e.g., electronic or manual blood pressure device, blood sugar meter). Meanwhile, others are taken automatically through common commercial wearable smart devices (e.g., smartwatch/band). Based on this hypothetical scenario, please answer the following questions.

Question: “How useful do you find the before mentioned information in your clinical assessment in the context of general monitoring of your patient’s health? Please rate: from 1—5 (5 = Very useful)”

**Figure 4:**
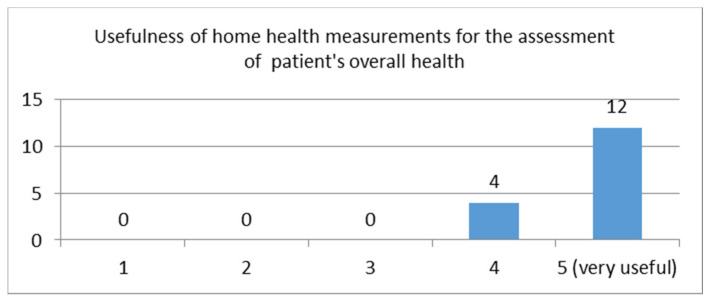
Distribution of rating scores for the usefulness of information captured by medical and non-medical devices for the clinical assessment of a patient’s overall health.

This question received a very high average score of 4.75 (STDEV = 0.43). The distribution of the scores is depicted in Figure 4. The results show that healthcare professionals are very interested in seeing results of health measurements taken at home, whether it is from common medical devices or from modern devices such as smartwatches/bands. The answers also implied that such measurements would be taken into consideration in the clinical of an initial assessment.

Question: “How interested would you be in having direct access to the patient’s measurement results from such devices? Please rate from 1–5 (very)”.

**Figure 5:**
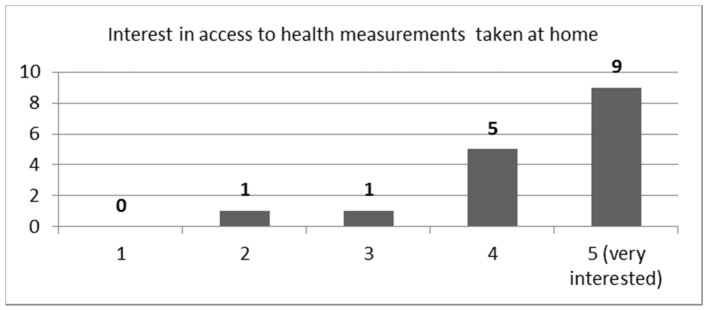
Distribution of rating scores for interest in having direct access to health measurement results from such devices.

An equally high average rating score of 4.3 (STDEV = 0.85) was received in the follow-up question of whether they would be interested in having direct viewing access to the results of such measurements from such devices. The distribution of scores is depicted in Figure 5. One physician in the notes said that he would like to first see the device used for the measurements before taking them into consideration.

Question: “Which of the following available measurements would you be interested in monitoring through the hypothetical electronic system? Check all that apply.”

Other important information gathered from this questionnaire had to do with which type of metrics they found important. The results are shown in Table 10 below.

An additional type of metrics they would like to see in a monitoring system were the following: water intake, body weight, falls (i.e., times person fell), number of cigarettes smoked (for smokers), sleep, and number of steps taken on a daily basis (Table 11). These results will be explored in future versions of the MHW system.

### 5.3. Questionnaire Results—Caregivers

Ten caregivers participated in the study and answered the third questionnaire. Of these 10, 7 were caregivers of a relative, 2 were home assistants, and 1 was working as a volunteer in a program called “Help at Home” (Table 12).

Question: “What type of assistance do you provide for the elderly person? Select all that apply.”

This user group was asked what type of assistance they provided to the person they cared for. They selected multiple services from the provided list. The available options and the number of times each option was selected are shown in Table 13 below.

Question: “What type of medical devices does the elderly person you assist use at home?”

In this question, all 10 said that they use a blood pressure meter, 5 said they also use an oximeter, and 2 a blood glucose meter.

Question: “Does the elderly person you care for use the medical devices on his/her or with assistance?”

In this question, 9 out of 10 of the caregivers said that the elderly persons they care for cannot use the devices on their own. This information revealed the importance of supporting automatic health measurements taken from various sensors. In addition, it stressed the importance of the end application to be designed in a user-friendly manner taking into consideration older adults’ characteristics.

Question: “How do you or the elderly person you care for keep a record of health measurements results?”

In this question, eight of them said that the recording of the results is performed manually on a piece of paper and two that it is stored in the device automatically.

Hypothetical scenario questions

The following hypothetical scenario was given to this user group: “Imagine that the elderly person you care for has an electronic program through which he/she can input the results of health measurements in the context of his/her monitoring of the overall health. Some of the measurements are taken through common medical devices (e.g., electronic or manual blood pressure device, blood sugar meter). Meanwhile, others are taken automatically through common commercial wearable smart devices (e.g., smartwatch/band). Based on this hypothetical scenario, please answer the following questions)”.

Question: “how useful you think such measurements are in the context of the care you provide to the elderly person?”

**Figure 6:**
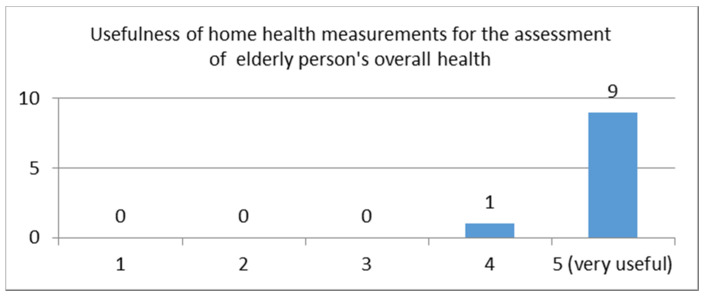
Distribution of rating scores given by caregivers for the usefulness of information captured by medical and non-medical devices in the context of elderly care.

This question received a very high average rating score of 4.9 (STDEV = 0.3), showing that just like the health care professionals, this user group also finds health measurements taken at home from medical or non-medical devices very useful in the context of care provided to elderly people. The distribution of the rating scores is depicted in Figure 6.

Question: “Which of the following available measurements would you be interested in monitoring through the system? Check all that apply.”

Other important information gathered from this questionnaire had to do with which type of metrics they found important. Blood pressure was selected by all, followed by stress level, and then heart rate, and blood oxygen (Table 14).

### 5.4. Semi-Structured Interviews Results

Semi-instructed interviews were held with two physicians during the requirements phase to extract more detailed information on their attitude towards the importance of vital signs monitoring at home. The interview included questions used in the questionnaire and additional questions on their overall impression of the MHW concept. One of the main questions in the interview was whether they would trust measurements from home devices, medically certified or not, in their initial assessment of an elderly person. Both physicians said that they would definitely take them into consideration in the context of the initial interview with their patient. The physicians said that measurements taken at home always have the risk of not being accurate as it has to do with how and when the person takes them, the device used, etc. Therefore, they have to be assessed in combination with other medical information. However, when measurements are continuous, it is easier to distinguish if an off value is just a fluke incident, a user error or an indication of a possible health problem. Overall, they both acknowledged the need to encourage self-monitoring of health, especially in the elderly population to reduce the need for hospital visits when possible. They were also positive in the idea of a comprehensive system that would allow the remote monitoring of elderly people when needed such as in the context of after-hospital care.

In addition to the above, two semi-structured interviews were held with two occupational therapists with great experience in working with elderly people. The goal of these interviews was to gather information on elderly people’s characteristics and their impression of how MHW could fit in their daily environment. During the interviews, the participants were presented with the MHW concept and asked for their opinion on its usefulness and possible applicability. They both stressed that a lot of elderly people receive support for the management of their health from people of their close environment, relatives, neighbours, or friends. However, these non-professional caregivers do not always live on the same premises as the elderly people and may not have everyday contact with them. In this context, both therapists said that the MHW system would be useful as a remote health monitoring system, which could allow them to have an overview of health status. However, they also stressed that it is extremely important for elderly people to have control of the decision making as to what data they would like the system to track, whom they want to share this information with, and under what conditions.

### 5.5. Discussion of Results

Overall, information gathered from the above questionnaires and the interviews played an integral role in the design of the functional characteristics of the mobile application concerning the type of metrics, the frequency of the notification, the parameters for sharing the data, etc.

Analysis of the requirements based on the results of this study resulted in the following main highlights:

1. Older adults and especially those who live independently in their own homes are not foreign to contemporary mobile devices (smartphones and tablets) or to the use of social media and other communication applications.
2. Older adults are not particularly meticulous in keeping a track record of their health measurements.
3. There are diverse needs and preferences when it comes to whom the elderly people want to share their health data with, making it even more important for them to have control in the decision-making.
4. Healthcare professionals and caregivers alike are positive about the notion of using vital signs readings collected by commercially available wearables, to support self-monitoring of basic health parameters for elderly people.
5. Healthcare professionals are positive towards considering vital signs readings from commercially available wearables in the context of an initial assessment of the health status of the elderly person.
6. Independence/self-efficiency and good health are of great importance in regards to quality of life.

## 6. MyHealthWatcher System

This section presents the design and development process of the proposed MHW solution and its sub-components. This includes the following: the architectural structure; the mobile application for elderly people; the sound processing subsystem employed by the mobile application; and the web-based application for healthcare professionals and caregivers.

### 6.1. Architecture

The design process of the MHW system architecture was based on concepts for subdividing a system into functional elements presented in [44]. Following this approach, we started from the broader analysis of the elements of the MHW system on a conceptual level based on the requirements collected from the previous stages of the project. Then, we moved to the conceptual architecture analysis, which specifies all the software and hardware components that together will form the foundation of the MHW system. In our case, these include the mobile device that the elderly users will need to have in order to interact with the MHW mobile application, the sensors that will feed collected data to the mobile application, the speech and sound detection system for recognition of pathological factors in the acquired sound signals, the web-based monitoring application for the healthcare professionals and caregivers, and the databases that will store sensor data and other relevant information. From the conceptual architecture analysis, we moved to the design of the High-Level Architecture, which provides a more detailed analysis of the software arrangement of the envisioned system. It includes the previously identified conceptual modules, their respective clustered functional components, as well as the external data collected from the sensors used in the system. Within the high-level architecture analysis, the system’s structures and its exposable attributes along with their explicit properties are determined.

From the High-Level Architecture, we moved to the Static Structure, which refers mainly to the design of the architectural elements of the system (objects, components) and how they all fit together internally. It constitutes the software that details the architecture analysis of functional components to be implemented. More specifically, the static arrangement of the architectural elements depends on the actual context of use and provides information such as associations, relationships, or connectivity among them. For instance, relationships define how data items (either input or outputs) are linked to another one. For hardware, the relationships provide the required physical interconnections between the hardware components and sub-systems comprising the overall system.

Lastly, the process ended with the definition of the Dynamic Structure of the system, which illustrates how the system actually works during its use, depending on the various use cases and scenarios of use previously defined in the project and the way each software functional component acts within them. It constitutes an evaluation mechanism for the determined functional components by applying them to a specific use case.

### 6.2. Architectural Development Process

**Figure 7:**
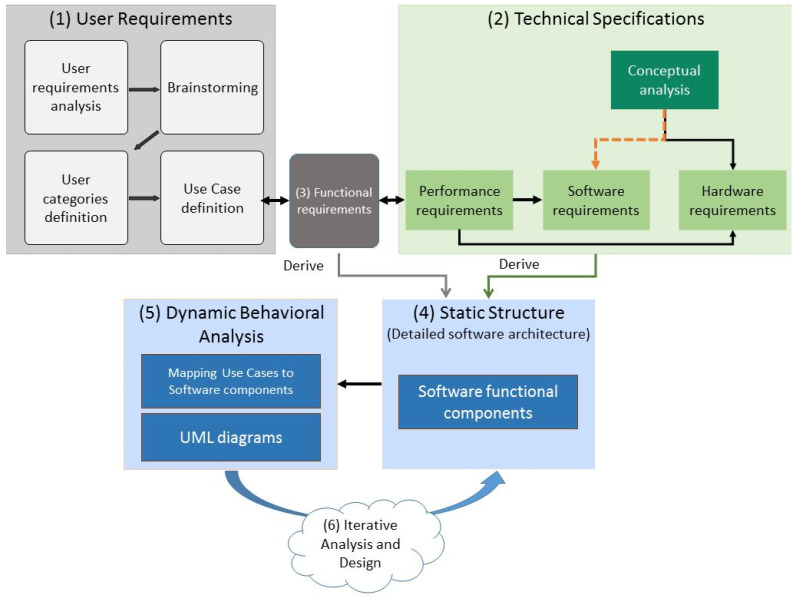
Architectural elements design process.

The process followed to design the MHW architectural elements and to mitigate from the high-level architecture to the static and dynamic structure of the system is depicted in Figure 7 and described below:

**Step 1**: Definition of user requirements. This step utilised the information collected in Stages 1 and 2 of the overall methodology (described in Section 3), to define the user requirements and their respective use cases, possible solutions to specific problems, and limitations.

**Step 2**: Technical specifications. A conceptual decomposition of the system into the fundamental structures in terms of hardware and software requirements was performed. The latter was achieved by taking into consideration the performance requirements and the conceptual analysis of the software requirements.

**Step 3**: Functional requirements. The interface between user requirements and technical specifications comprises the system functional requirements. In more detail, each use case was interpreted as a sequence of steps to be completed by the software and hardware to fulfil a certain task. This assists in the determination of the essential functionalities that the system should retain.

**Step 4**: Static structure. The static model of the MHW system constitutes a detailed analysis of the essential software parts to be implemented. It comprises the set of algorithmic routines to be developed within the lifespan of the project. In addition, the static model holds a detailed description and definition for each software functional component providing explicit details about their functionality, their in-between dependencies, their operation requirements as well as their indicative performance requirements.

**Step 5**: Dynamic behavioural analysis. Once the static model of the MHW system was determined, its functionality was evaluated on the dynamic model. In this process, the functionality of the system was examined by assessing its overall behaviour while running various scenarios of use, as well as the behaviour of each of its modules.

**Step 6**: In the last step, the procedures described in steps 3 and 4 were reiterated to make sure that no functionality was missing and to improve upon the established functional components before concluding on a valid and globally descriptive static model.

### 6.3. High-Level Architecture

**Figure 8:**
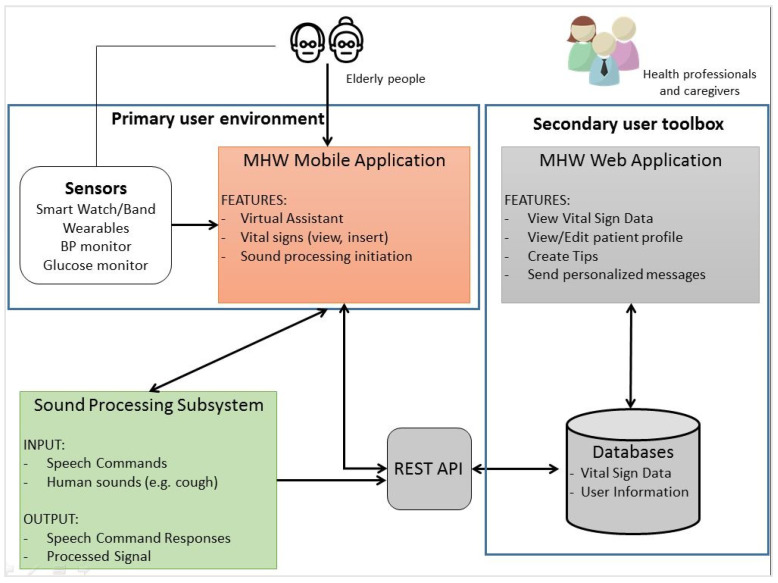
High-level architecture.

Figure 8 presents the functional modules of the architecture of the system. This includes the primary user environment (mobile application and sensors), the secondary user toolbox (Web-based application and the databases), and the sound processing subsystem, which handles the speech recognition and the identification of pathological factors in the sound signal. Their components are described separately in the next paragraphs.

### 6.4. MHW Mobile Application

The MHW mobile application is part of the primary user environment as depicted in Figure 8. It consists of the user interfaces through which the elderly people (primary user group) can interact with the system and the connected sensors that track selected vital signs. Through the mobile application the user can perform tasks such as: viewing their current overall health status based on the latest health measurements taken by connected sensors; inserting health data manually from other medical devices not connected to the mobile application; viewing personalised tips and articles on managing chronic ailments shared to them by their healthcare professional; and, receiving personalised messages from the latter. Furthermore, through the application, the elderly people can specify their list of contacts, which consists of the healthcare professionals and other caregivers whom they wish to have access to their health data. The application also includes a virtual assistant in the form of a human-like avatar to support the user interaction with the mobile application. This component is described in more detail in the next section.

**Figure 9:**
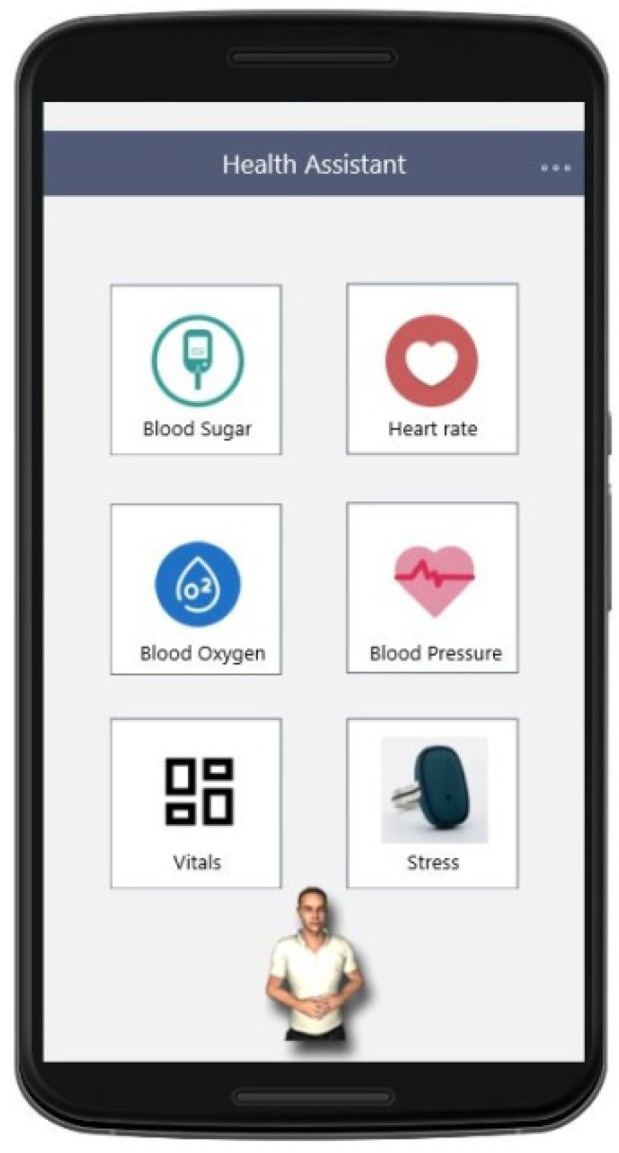
Home screen initial design prototype.

**Figure 10:**
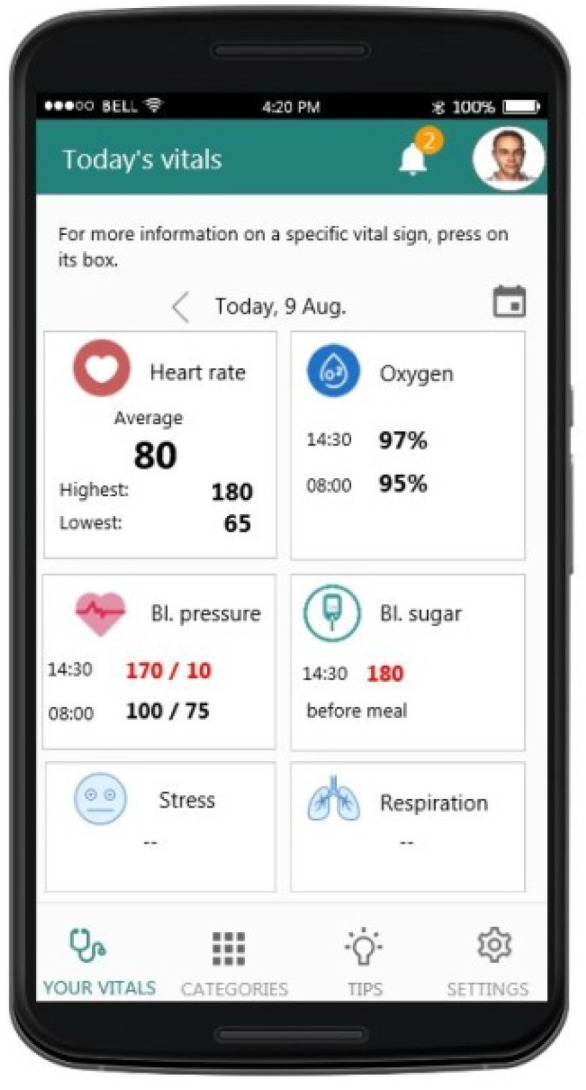
Home screen after expert review. This version was used for the user-based evaluation.

**Figure 11:**
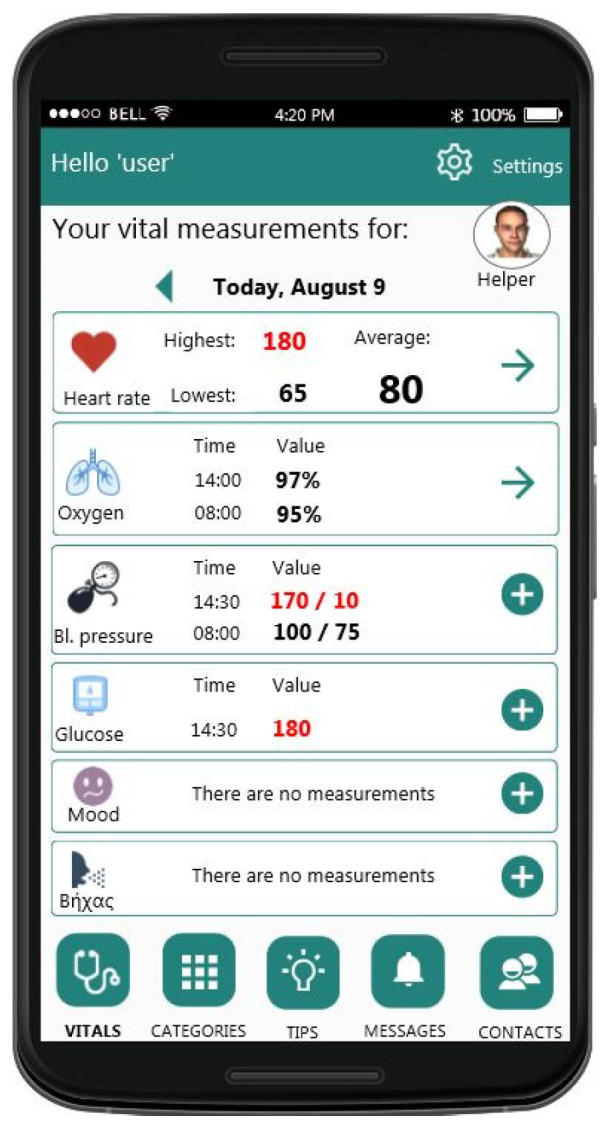
Home screen after applied changes based on the user-based evaluation results.

As mentioned in Section 3, the design of the user interfaces (UIs) of the mobile application was built based on the requirements collected at the early stages of the project and went through several design/evaluation iterations. In Figure 9, Figure 10 and Figure 11, an example of the progression of the design prototypes for the home page of the logged-in user is shown, based on feedback received during each design evaluation iteration (expert and user-based). All the UIs presented in this paper were translated from the Greek language for presentation purposes. Additionally, any health data depicted on the UIs are mock-ups that are used for presentation purposes only. In the first version, the user had to click on a vital icon to view the collected data for that day. The design was cleaner, but the user could not easily see all the vitals on one screen. As the expert reviewers pointed-out, the constant back and forth to read all vitals is cumbersome for the user and creates unnecessary steps in the process, an issue which was addressed in the second version. In the last one, the graphical treatment of navigation buttons is more prominent than its previous version, as the elderly users noted that they did not look like buttons. They also suggested adding arrows and “add” buttons (indicated by a + icon) in the same colour to know where to press to select a vital category.

**Figure 12:**
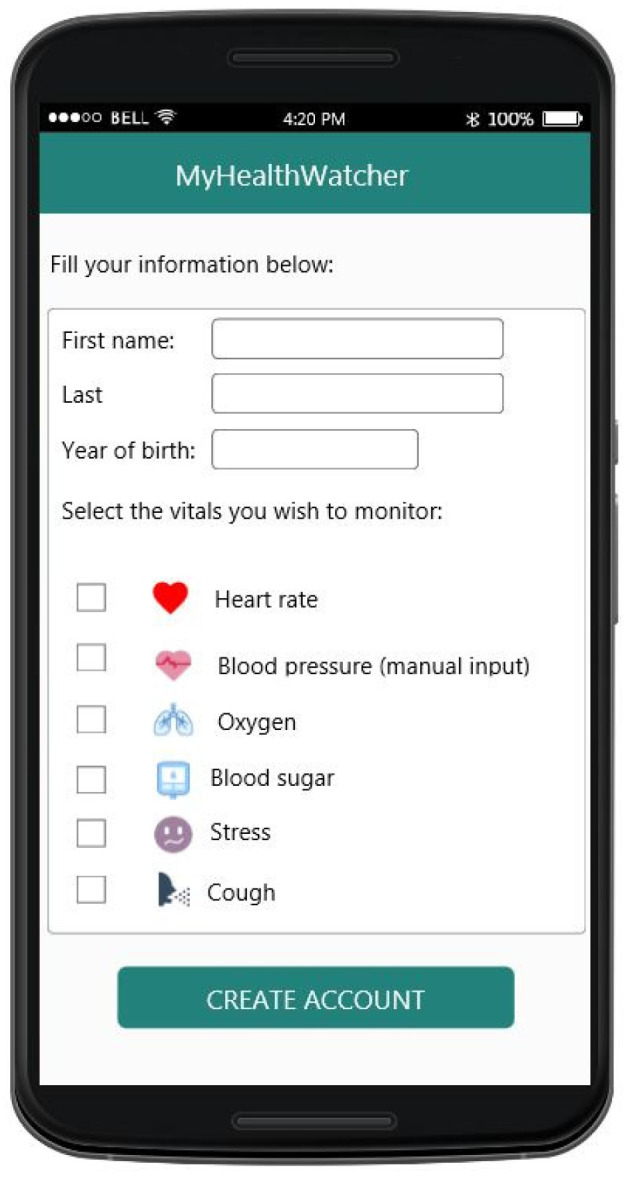
Account information screen, where the user specifies which vital signs he/she wants to monitor.

As it became apparent from the requirements analysis, each person experiences ageing differently, therefore it is important for the user to be able to select which vital signs he/she wants to track in relation to their health condition. In the mobile application, the user can specify which vitals he/she wishes to monitor from the account information UI (Figure 12). Furthermore, this information can be changed as needed at any point.

**Figure 13:**
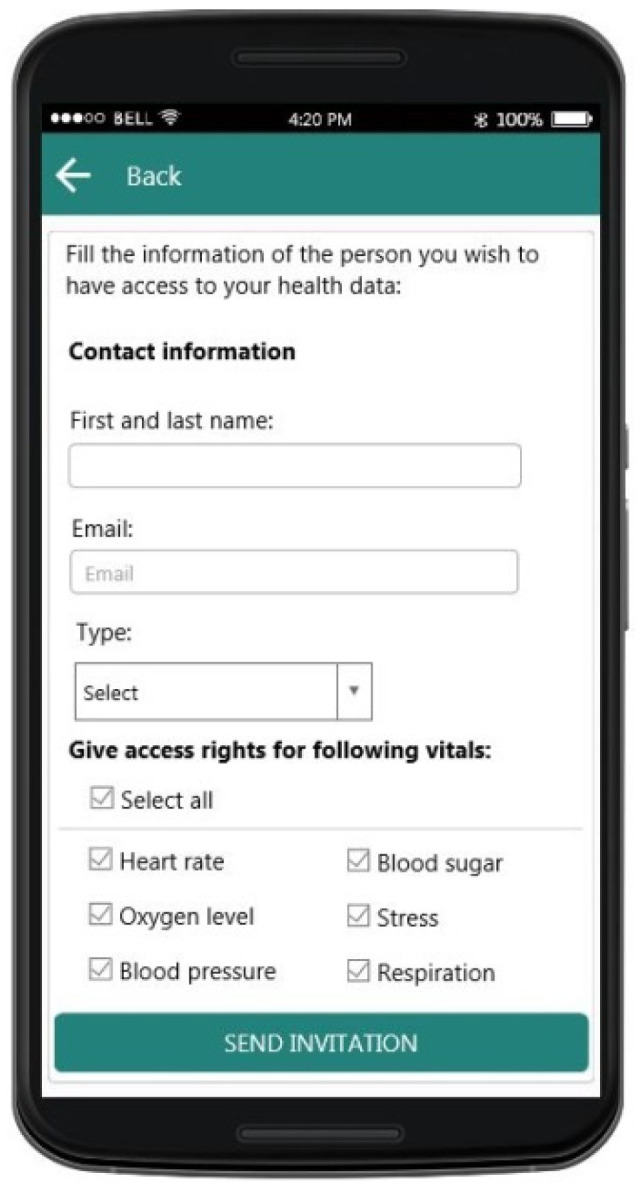
Create a contact invitation screen. The user selects the vital signs the invited contact will have access rights to.

Another crucial point that was revealed during the user requirements analysis phase was the fact that elderly people would like to have control as to who has access rights to their data and to which categories of data that person has access right to. To address this requirement, in the application the user can create their own list of contacts who will be able to have access rights to their health data. To create a contact, the user sends an invitation to that person and in that invitation, the user specifies the type of data they will be able to have access to (Figure 13). Furthermore, the user can change the previously mentioned options as needed at any point.

#### 6.4.1. Virtual Assistant

**Figure 14:**
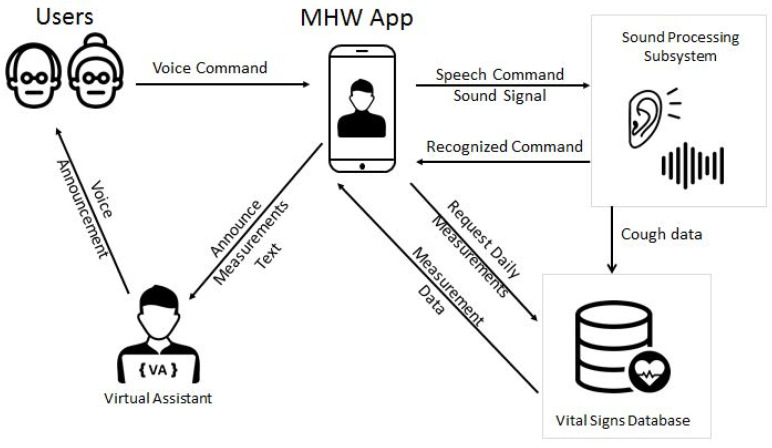
Avatar communication workflow.

The virtual assistant’s role in the system is to enhance the user experience while interacting with the system. The assistant is offered as an optional service to the application’s user and is always available through its portrait icon on every application screen. When the user accesses the service, the virtual assistant is shown as an animated three-dimensional representation of a human-like avatar. The virtual assistant’s screen design is deliberately a bit different from the rest of the application, to make it clearer that it is a different interface. The virtual assistant is able to simultaneously announce and show in text various information. The user has quick access to their latest measurements and alerts using buttons that prompt the assistant to retrieve relevant information and announce it. For the rest of the functionalities, the users can press the “microphone” button and then speak the appropriate speech command to the assistant (e.g., “tell me my oxygen saturation” or just “oxygen”). In Figure 14, the workflow of how the avatar communicates with the rest of the system is depicted in

The first iteration of the virtual assistant avatar was designed using Adobe Fuse CC, a quick and intuitive model creator with built-in models. The application offers a range of body types, faces with detailed customization of facial features and a selection of clothes. A full-body model was created and exported for rigging using the compatible Mixamo [45] service. This service also includes a large variety of predefined—animations that are compatible with the prepared model. A number of them were selected to be used by the virtual assistant. The exported 3D model was inserted in a Unity scene along with a proper background and was integrated with a lip-synchronisation library [46]. The set of animations were hooked to the model using a script in order to be able to initiate them using the appropriate command. In the same way, “speak” commands were scripted to correspond to appropriate lip movements following the supplied text. The completed Unity scene was then imported into the native Android MHW application. By integrating the scene inside an Android graphics surface, it was possible to create a user interface with the virtual assistant covering the background and other functionalities on top of it, such as buttons around the assistant or a speech bubble that shows the text currently spoken.

The interoperability layer between Unity and native Android development allows simple method invocation and parameters, using primitive variable types. The mobile application has access to a basic application programming interface which allows basic functions such as starting or stopping a supported animation to be performed by calling the appropriate Unity script and supplying the parameters in order.

The selection of Unity and the implementation of an interoperability layer were intentional, so as to minimise the parts of the overall systems that are developed for specific technologies and devices. Through this direction, the avatar part, which requires heavy development, is cross-platform, and the mobile app part can be easily ported to other development platforms.

A more sophisticated approach was taken for implementing text-to-speech. Unity models can offer lip-synchronisation capabilities, but the text-to-speech system resides on the mobile application part. The solution was to encode the sound signal from the text-to-speech system and send it programmatically through the interoperability layer to the Unity module, which then plays it with correct lip-synchronisation.

#### 6.4.2. Sensors

There are a number of compatible sensors that can connect to the mobile device enabling the mobile application to extract their measurements automatically and periodically.

Based on the user requirements analysis, the sensors to be used in the system should have one or more of the following characteristics and functionalities:

- Heart Rate monitoring
- Blood Pressure monitoring
- SpO2 (peripheral capillary oxygen saturation) measurement, preferably without a finger sensor
- Non-intrusive Galvanic Skin Response measurements
- Blood glucose level monitoring
- Low cost
- Wireless connectivity (Bluetooth-enabled)
- Daily use without hampering mobility
- Easy software toolkit to extract data from a third-party application
- High and enduring market availability

To satisfy the above requirements and narrow the search for suitable sensors, all finger oximeters and devices that use intrusive methods (e.g., electrodes) for measuring galvanic skin response were rejected in favour of wrist wearables and rings. No affordable and market available galvanic skin response wrist wearable was found, therefore a ring sensor was selected for this purpose, the Moodmetric smart ring [47]. The ring measures the reactions of the autonomic nervous system with an accuracy of a laboratory device and requires a Bluetooth connection to send the data to the mobile device. The measurement registers emotional and cognitive load in real-time and assigns it to a simplified 1–100 scale. According to the research posted on the manufacturer’s website, following two weeks of measurements, daily average values over 50 can be interpreted as stressful and might require attention

For blood pressure monitoring, it was decided that it would be better not to impose any specific device or range of devices to the user and let them use their own blood pressure monitor, inputting the results in the application manually, since users usually already own a legacy blood pressure monitor without connectivity. For the same reason and the fact that not all the users will have to monitor their blood glucose level, we choose the manual data input for the monitoring of the blood glucose level. For heart rate and SpO2, two solutions from Garmin were selected, the Vivosmart 4 and the Fēnix 5X Plus. Both are suitable for the system’s needs, with the Vivosmart 4 being the most affordable option and the Fēnix 5X Plus with more capabilities as a smartwatch such as apps, but at a rather high price range.

Garmin’s Standard Health SDK was used to provide direct access to the sensor’s data from the mobile application without requiring another external application or creating an account to access the data on a proprietary server. Periodic measurements of heart rate and oxygen saturation are available and are sent via Bluetooth to the mobile device to be immediately stored in the vital signs database. Other available measurements include steps, calories burned, floors climbed, accelerometer data and body battery energy monitoring. Some of them could be considered for a future version of the system.

### 6.5. Sound Processing Subsystem

The MHW system utilises the microphone embedded in the user’s mobile device in order to capture, upon request, sound signals produced by the user. As shown in Figure 8, the compressed sound signals are sent to a server that offers two basic services, speech recognition and the identification of pathological factors in the sound signal.

The speech recognition service is used for voice interaction with the Virtual Assistant component of the mobile application as described in Section 6.4.1. It is based on a Hidden Markov Model-based speech recognizer developed by European Media Laboratory [48]. The acoustic model was trained upon 140 h of manually annotated speech, using data that were gathered from Greek native speakers. It is noted that this is a general-purpose acoustic model and no actions were taken in order to adapt the acoustic model to the particularities of speech from elderly users. On the other hand, a limited-vocabulary language model was specially designed for the needs of the MHW project, with the main goal to facilitate the communication of the mobile application user with the virtual assistant. In particular, the developed language model, in combination with the SR system workflow, allows the end-user to use almost natural language, without the need to remember specific commands. For example, different speech commands are allowed to correspond to the same functionality of the application, e.g., the commands “heart rate”, “tell me my heart rate” or “how much is my heart rate? ” correspond to the same functionality regarding this health indicator. This way, the user does not have to remember specific commands and the communication with the system is carried out intuitively using almost natural language.

The second service employs acoustic signal processing with the aim to detect coughing events. The cough detection service is initiated from the mobile application and operates during a user-defined period of time (e.g., during sleep time). The recorded audio data are processed offline at the server and relevant metadata, such as the number of detected cough events and their temporal location in the recording, is stored in the database. Moreover, portions of the original recording classified as cough are stored as audio files in the database so that they can be played-back upon request from a professional expert (e.g., a medical assistant). The next paragraphs provide some technical details regarding the implementation.

The cough detection system combines an acoustic onset detector and a Deep Neural Network classifier at the back-end. This pipeline is motivated by the observation that at the explosive phase of the cough, the sudden release of air from the lungs leads to a rapid increase in the acoustic energy. This variation can be efficiently detected in the acoustic signal with the utilization of onset detection methods [49].

**Figure 15:**
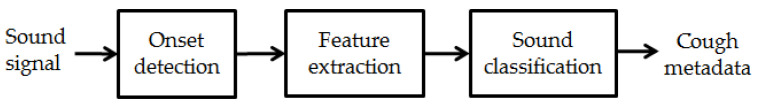
Pipeline used for detecting cough events in the sound signal.

Consequently, the onset detection stage may significantly reduce the amount of audio data that has to be presented to the classifier, at the risk of course of missing some cough events. When onset is detected, acoustic data are extracted along a short temporal segment that starts 0.125 s before the onset and ends 0.5 s after it. For each 0.625 s duration audio segment, the corresponding log-mel spectrogram is produced (middle box of Figure 15), which represents the acoustic feature input to the DNN classifier.

Regarding the DNN classifier (right box in Figure 15), the Long Short Term Memory (LSTM) architecture was used as it was found to perform better compared to other DNN architectures. Specifically, two LSTM layers of 256 units each were used, followed by one fully connected layer of 64 units and finally a dropout layer with 0.3 probability and an output softmax layer.

The interested reader is referred to [50] for a more detailed description of the cough detection system as well as of the data collection process.

The key to the successful operation of both the speech recognition and the cough detection system is the use of multi-condition training, i.e., training the recognizers with acoustic data captured in varying acoustic conditions and with different recording devices. This is important in order to make the systems robust to noisy and/or reverberant acoustic conditions and also, in order to guarantee a stable recognition performance across different electronic devices with different microphones and sound acquisition characteristics in general.

### 6.6. Web-Based Application

The web-based application is part of the secondary user toolbox as depicted in Figure 8. The Web application is built using technologies from Microsoft’s.Net Core family of interoperating technologies, including ASP.Net Core MVC and Entity Framework Core. The framework includes its own webserver to service requests, however, in order to enable more advanced features such as load balancing and secure connections, nginx [51] is used as a reverse proxy. The website is hosted on an Ubuntu Linux 18.04 Server. The same platform also publishes a REST API that acts as a bridge between the mobile application and the databases. The API utilizes JSON Web Token [52] features for authenticating the mobile application users and the same token is used for authorizing them to access data relevant to the application functionalities, such as retrieving messages, storing and retrieving measurements, adding contacts and others.

As mentioned earlier in the paper, the Web-based application provides the interface between the healthcare professional and the elderly person whose overall health is monitored. The connection between the elderly person and the healthcare professionals is achieved in two ways. In the first way, the connection is initiated by the elderly person through the mobile application by sending an invitation to their healthcare professional or caregiver via an email and asks them if they want to monitor their health. In this case, the healthcare professional from the invitation email, he/she will select the provided link which will lead them to the Web-based application account page. Once the user creates the account and accepts the invitation, the connection between the two applications is established. In the second way, the connection is initiated by the healthcare professional, by sending an invitation to the elderly person who receives it in the mobile application previously installed in their mobile device.

The structure of the web application UI is simple. The main navigation menu bar is situated at the top of the application with options to the main categories of content, e.g., dashboard page (homepage), contacts page, alerts page, messages page, and tips page. The design of the UIs went through a couple of iterations and the designed prototypes were incrementally improved based on feedback received by two HCI usability experts in the context of expert-based inspections and by domain experts (i.e., two invited physicians who participated in design discussions). Once the designs matured, a preliminary user-based evaluation was conducted with the participation of three physicians. The results of these are reported in Section 7.2 of this paper.

A brief description of each of the application’s main features is given below as well as screenshots of the currently implemented UI.

**Figure 16:**
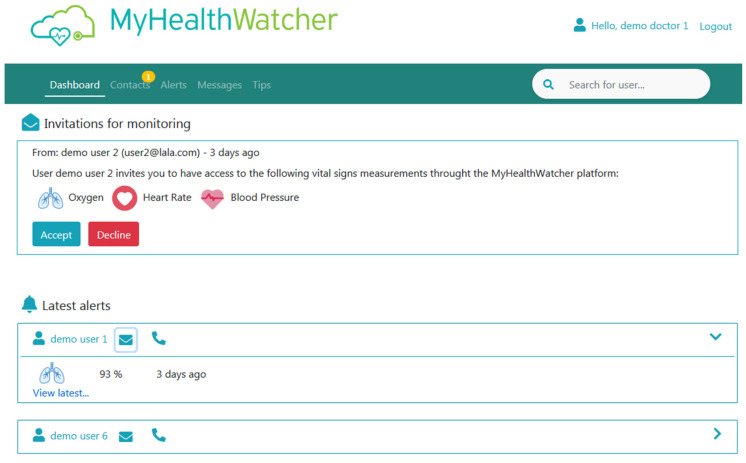
Screenshot of the UI for the Dashboard page—current implementation.

Dashboard view: This page (Figure 16) works as the homepage of the application and shows the most current and important information. This includes invitation messages from primary users of the mobile application that are asking the professional to monitor their health and a list of the latest medical alerts caused by the detection of values outside the normal range (per user). From here the user can perform quick tasks such as accept/decline an invitation, click on the name of a patient with medical alerts to visit his/her profile data, click on the message icon to send him/her an immediate message, and access their phone number. Furthermore, on the top of the application appears the global navigation menu with option for navigating to the main content areas of the application (e.g., Contacts list, Alerts lists (aggregated data for all monitored patients), Messages (all messages sent to any of monitored patients), and Tips list (list of created articles and health management content).

**Figure 17:**
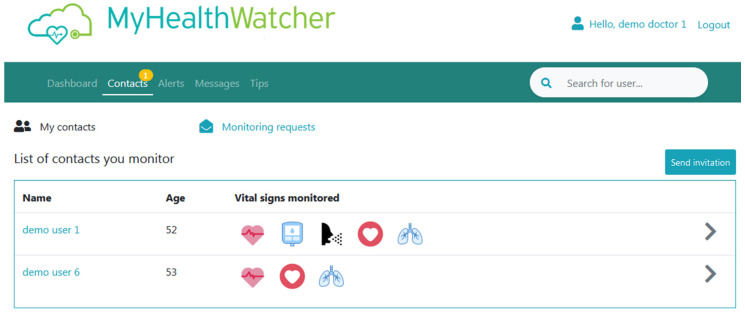
Screenshot of Contacts main page—current UI implementation.

**Figure 18:**
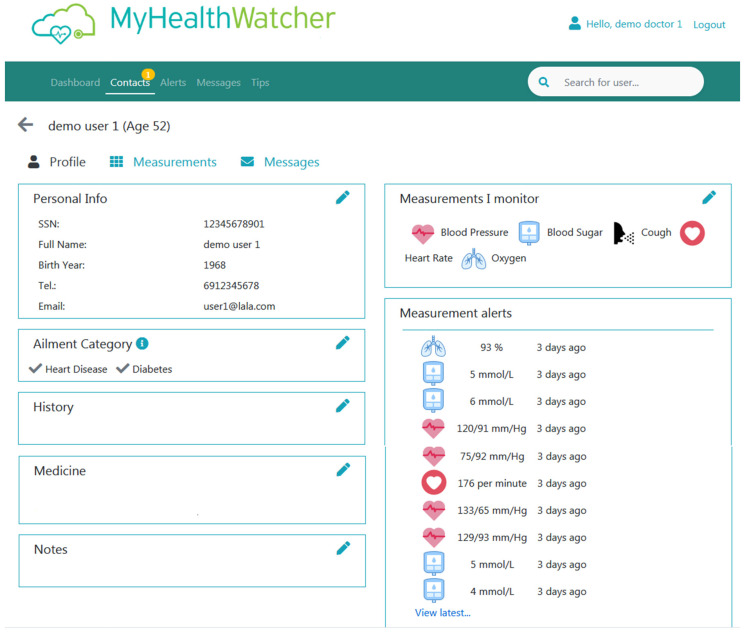
Screenshot of selected patient’s profile view page—current UI implementation.

Contacts view: From this page (Figure 17) the user has access to the list of all the people that he/she is monitoring along with the vital signs that he/she monitoring for each person. The user can click on the name of any person listed here to access their profile page (Figure 18) or the right arrow button to go to the measurements’ page of that user.

Profile pages: This page shows the profile information of a selected patient. From here, the user can perform basic tasks such as view personal information, edit the patient’s information, and add notes about their medicine regime, health history, and others. From this page, the healthcare professional or caregiver can also see all the “alerts” he/she has received for that particular patient in chronological order. Lastly, the user can navigate to the “Measurements” page that shows all the results of health data captured by the sensors of the selected elderly patient. Lastly, from the “Messages” menu option can view the messages he/she has sent to that particular patient.

Messages: This page shows a list of messages sent to patients.

**Figure 19:**
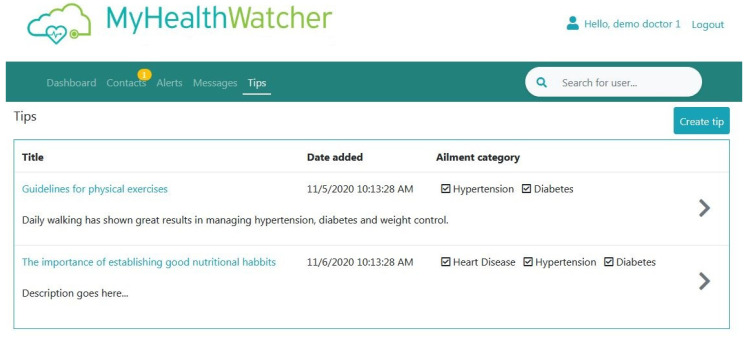
Screenshot of Tips view page—current UI implementation.

Tips: This page shows a list of general health-related content created by the user for his/her patients. For each “Tip”, the user can check the type of ailment it regards to, so that only patients that have this ailment will be able to view it through their mobile application (Figure 19).

#### Databases

The system requires the storage of vital signs data and other information for the mobile and web applications to function. The TimescaleDB [53] extension for the open-source relational database management system PostgreSQL was used to accommodate the system’s databases. The extension supports relational SQL queries and PostgreSQL compatible operations on hypertables, which are optimised tables for time series data. This ensures fast and reliable queries for the multitude of sensor data stored periodically by the sensors connected to the primary users’ mobile application. Using a database extension instead of a separate database management system enables all the relevant data to be stored and accessed in a uniform way. The database was installed in an Ubuntu Linux 18.04 Server.

Apart from vital signs data stored in hypertables, the database also stores the following:

- User account and profiles (e.g., username, contact details)
- List of users (e.g., patients) monitored by the secondary users along with information on which vital signs are monitored with the primary users’ consent
- Upper and lower thresholds for every measurement type, used to create alerts
- Abnormal measurement value alerts
- Health professional tips
- Messages sent to primary users
- Invitations to add as contact, from primary to the secondary user and vice versa
- Additional information that health professionals might keep for their patients (e.g., history, medicine and notes)

## 7. Evaluation Results

As mentioned in the Methodology Section of this paper, iterative evaluations were performed during the design process of the mobile and the web application of the system. Initially, the evaluations were performed in the format of expert-based evaluations by HCI usability experts. As the UIs became more concrete, we conducted a user-based evaluation with representative users of the two user groups, elderly people and healthcare professionals to test usability aspects of the UIs and overall impression of the system. For the needs of these evaluations, specialty software for prototype evaluations was used to transform static design mock-ups into fully interacting working prototypes. This software allows the interlinking of the prototype screens in a way that simulates real interaction. This way the user can use the prototype as if it were fully functional. The working prototypes were then loaded on mobile devices for the evaluation of the mobile application and on the browser for the evaluation of the Web-based application.

Furthermore, speech recognition and the cough detection components of the sound processing subsystem were evaluated separately in terms of performance using objective metrics. The process and the results of the above evaluations are presented separately in the subsections that follow.

### 7.1. Mobile Application Preliminary Evaluation

The evaluation with elderly users was performed in the context of a participatory evaluation workshop which was put together to receive general impressions on the overall MHW system, feedback on the design and functionality of the mobile application prototype and collect ideas and suggestions on how to improve it. A total of 16 elderly people and 2 occupational therapists who provided assistance when necessary participated in the design workshop. Taking advantage of the presence of so many representative users in the workshop, a collaborative evaluation activity was included in the program to help us identify usability problems in the design of the UIs. Additionally, given the fact that the participants had little experience with smartphones and almost no experience, with the exception of two users, of using complex mobile applications, the collaborative evaluation technique [54] helped to alleviate any stress or fear of having to use an unknown-to-them technology. The fact that the participants knew each other made them feel at ease and more confident in their participation.

At the beginning of the workshop and prior to any activities, participants were introduced to the aims and objectives of the workshop. In particular, the process that would take place was explained, as well as issues related to the anonymisation of their data, as described in the Informed Consent Form, which was signed prior to the experiment. Next, a short presentation was given regarding the MHW system, its goals, and inspirations. Furthermore, in the presentation, the purpose of the participatory workshops and evaluation activity was explained and the importance of their input and insight was emphasised. It was also explained to the participants that the goal was to evaluate the design and performance of the prototype and in no way to evaluate or assess their performance in using it. Special emphasis was also given to the fact that any health data displayed on the application were not real and were entered by us for the purposes of the activity. We also explained that their participation was voluntary and that they had the right to stop at any time and for any reason without any consequences. We also gave to each participant a user-consent form at the beginning of the workshop to sign after explaining to them the type of data that we were going to collect, how we were going to use it, and the fact that the results would be processed and reported in an anonymised manner to safeguard their identity.

For the context of the activity, we employed the use of personas (fictitious characters) [55] and role-playing [56]. We also constructed a fictitious scenario of use to help the participants understand the context of the use of the mobile application. Based on the scenario, the participants were asked to play the role of the close friend of the fictitious persona, Mrs Evgenia, an active and independent living 70-year-old woman, who was given a smartwatch band and a wearable device that measures stress as a gift from her son after she had been diagnosed with cardiovascular disease to keep track of various health vital signs and share them with her physician. The son had also installed the MHW mobile application on Mrs Evgenia’s smartphone so that she can view her vitals and had demonstrated to her the basic functionality. Mrs Evgenia, who does not have any experience in such applications, asked for the help of a close friend (the participants) in using it. Assuming the role of Mrs Evgenia’s friend, the participants were asked to complete a series of basic tasks using the mobile device and the application prototype.

For the evaluation activity, the participants were divided into small groups of three (1 group of 4). Each group was given a mobile phone with the loaded working prototype of the application. When the evaluation started, the evaluation moderator presented each of the tasks one at a time and allowed a short period of time for the groups to complete. Five tasks were given to the groups to complete. Three more HCI experts were present in the room and observed the interactions between the members of each group while performing and made notes on comments, observations, and questions that occurred during the interaction with the prototype. At the end of each task, each group was asked to answer subsequent questions regarding their interaction and the information they saw and express any problems they encountered. At the end of the evaluation, the participants were allowed to explore the application on their own and were then asked to note, on one post-it note, five things that they really liked the most in the application, five things that they liked the least, and suggestions for new features.

Overall, the impression of the overall MHW system was positive. The participants understood the purpose of the mobile application and how it can help a person monitor certain health measurements. All groups were able to complete the tasks in the time given and gave the correct answer to the subsequent respective questions. From the observations made by the HCI experts who were monitoring and making notes of their interaction with the system the following problems were captured:

- The layout of the information displayed inside each vital container on the home screen of the measurements was confusing to some users. The difficulty was expressed in the Blood Pressure display of the time of the measurement. Based on this, the layout of the vitals’ containers was changed to a vertical presentation of the containers to allow more room for displaying all the information in a clear manner.
- The button label for Adding a measurement was not well understood. The users suggested using a verb “ADD” or an icon with the “+” sign. Based on this, distinct button icons (arrow for navigating to the respective screen and plus sign to navigate to screen for entering a measurement manually) were added inside the vitals’ containers.
- They had trouble distinguishing between “Alerts” and “Messages” which they thought were the same. Plus the alert icon was on the top of the screen, whereas the messages button was on the bottom, causing further confusion to the users. Based on this, the alerts and the messages were consolidated in the new design and they were all accessed from a single button at the bottom of the screen.
- Some users thought that the avatar icon was the picture of the physician. Once they opened the avatar UI, they confused the “microphone” icon with the “instructions” icon and were selecting them erroneously. Based on this, we are working on making the avatar UI environment different from the mobile application environment, so that it is more distinguishable. Overall, the Avatar section of the application will be evaluated again extensively once it is fully implemented and integrated into the system.
- From the comments written on the post-it notes at the end of the session, what seemed they liked best was the overall concept of the system and the fact that they would be able to track their health through the wearables. They also liked the fact that they could connect to their physician through it. As far as the UI traits that they found cumbersome were the font-size of the text, the colours of the menu buttons. As far as ideas for changes, the majority suggested the use of the plus and the arrow icons in all the boxes to make their actions clearer to the user. Additionally, some users said that they would like to see the picture of their doctor and to be able to talk to him directly.

### 7.2. Web-Based Application Preliminary Evaluation

In the evaluation of the web-based application, three healthcare professionals (physicians) participated in individual sessions. The process of this evaluation was the same as the one for the mobile application. The participants were first presented with the overall MHW system and its goals. The purpose of the evaluation was described and the process of the evaluation was explained to them. They were given user consent forms to sign and were told that their participation was voluntary and they had the right to stop at any time and for any reason. During the evaluation, they were given six tasks to complete sequentially and were asked to express their thinking process aloud (Think-aloud technique) during their interaction with the working version of the web application prototype. An HCI usability expert conducted these sessions and noted down the comments and insights expressed during each session.

The participants’ overall impression of the system was rather positive and all found it useful and applicable in a real-life scenario. They were all able to complete the tasks given with no observed problems and found the UIs and the structure of the information user-friendly. Comments were given in regards to the information stored in the profile of each patient and made suggestions for additional information they would have liked to be able to keep, such as adding a field for the healthcare professional to note the id number of the patience national insurance, a field to note the medication each patient is using, a button to connect to the national prescription system so that they could access that information easily, to add an option for receiving the alerts on an email address, and a text field to write the patient’s health history. Furthermore, one of the physicians suggested creating a common library of health-related articles so that each patient could see the articles that relate to their condition on their mobile phones. Another comment was made to not give the option of deleting a patient from the system but rather to archive their data, which will be examined in terms of compliance with the General Data Protection Regulation (EU 2016/679) before adopting it.

### 7.3. Evaluation of the Sound Processing Subsystem

#### 7.3.1. Evaluation of the Speech Recognition (SR) System

To have a realistic measure of the SR performance, a test set was created by recording the voice of elderly speakers in an indoor acoustic environment. A total of 100 different speech commands—covering several different functionalities of the MHW system—were composed with the use of the language model. These 100 commands were then split into four different groups of 25 commands each. Α single group of commands was then assigned to each subject who participated in the evaluation to read during the recordings.

The recordings took place in a relatively large office, with the participation of twelve female and four male elderly volunteers. Prior to their participation, the users were told the goals of the activity and how the collected data were going to be used for the purposes of this project. They were then given a user consent form to sign.

The sound was captured with the simultaneous use of two different devices and particularly

(i) A condenser lavalier microphone attached to the subject’s clothing. The lavalier microphone was connected to a professional sound card and the recordings were acquired in uncompressed (PCM) format.
(ii) The microphone embedded in a medium-cost smartphone device. The smartphone was placed on a table at a distance of at least 1 m from the subject’s mouth and the recording was acquired in uncompressed (PCM) format.

While the lavalier microphone guarantees better sound capturing conditions, the recordings produced with the smartphone device are more representative of the audio quality expected during the actual implementation of the MHW system.

Despite the difference in the recording quality, it may be seen from the results of Table 15 that both recording devices lead to almost the same recognition performance. In total, the number of false recognitions is 25 and 27 for the lavalier and the smartphone microphone respectively. From the 25 false recognitions with the lavalier microphone, two were caused by the inability of the system to associate the voice command to a given functionality and were thus classified as “unknown command”. The rest 23 mistakes were caused by recognizing false functionality. Correspondingly, the smartphone recordings produced 24 false recognitions and there were three utterances classified as “unknown command”. In total, the speech recognition accuracy is judged to be satisfactory for the needs of the MHW system.

#### 7.3.2. Evaluation of the Cough Detection System

To gain an impression on the cough detection performance, the performance of the onset detection and the sound classification stage were evaluated separately.

The process followed to evaluate the performance of the onset detection stage is the following. A total of 58 recordings containing cough events were randomly selected from the available dataset. A subject listened to these recordings in Praat and for each cough instant, marked the time interval starting a little before and ending a little after the cough onset. This way, 284 cough onsets were identified. Running the onset detection algorithm, the number of detected onsets that were located within these intervals were counted. In the end, the onset detection step spotted 275 out of the 285 cough onsets, achieving a detection rate of 96.8%. At the same time, 575 onsets that were detected by the algorithm were outside from the marked time intervals and were in most cases triggered by events that did not belong to the cough class.

As a first approach to evaluate the performance of the binary classifier, a four-fold cross-validation approach was designed, where each time one fold is treated as the validation set, and the method is fit on the remaining three folds. The cross-validation approach was designed in a way that ensured that portions from the same audio recording are never both in the training and in the testing set. To a large degree, this provides a realistic impression of the classifier performance on unseen recording conditions and on unseen recording devices. The results in terms of sensitivity and specificity, averaged across all four-folds, were 87.8% and 98.9% respectively. This implies that approximately 1 out of 10 cough events is missed by the DNN classifier, while approximately 1 out of 100 non-cough events is mistakenly classified as a cough event.

Finally, the classification performance was examined on the audio recordings that were acquired for the needs of evaluating the speech recognition performance, see Section 7.3.2). Applying onset detection on this set of audio recordings resulted in 4537 acoustic onsets, among which 27 were identified as cough events. The DNN classifier was able to spot 26 out of 27 cough events, which corresponds to a sensitivity of 96.3%. Moreover, 13 acoustic onsets not related to a cough event were mistakenly classified as cough, which corresponds to a specificity of 99.7%. The interested reader is referred to [50] for a more detailed description of the cough detection performance.

## 8. Discussion and Future Work

In this paper, we presented an adaptive system for monitoring the overall health and well-being of elderly people. To this end, the design and implementation methodology, the architecture and implementation structure of the system and the characteristics of its individual components were described in detail. Lastly, preliminary evaluation results were presented for the two main end applications, the mobile and the web-based application respectively as well as evaluation results for the sound processing sub-system.

Our experience so far indicates that following the HCD approach practices proved to be beneficial. The involvement of representative users throughout the design process gave us great insight into how people receive the concept of the MHW system, what features they want for the system to have, and how they expect it to behave and operate. It also helped us assess our initial assumptions on the relationship between elderly people and modern technologies. The overall positive attitude of the participants towards such a complex concept exceeded our expectations. We believe that the system can be used in diverse contexts of use and its use can be tailored to the needs of each user. Elderly people with more experience in using modern technologies would be able to use all the provided features and components of the system. Even elderly people who are not able to use a smartphone or a mobile application could still benefit from tracking their vitals through the connected sensors and allowing the monitoring exclusively to their physician or caregiver. On the side of the physicians, MHW can be viewed and used as a service for long-term monitoring of their patients. The aggregation of automatic health measurements in one application would give them a holistic initial assessment of the overall health of their patients.

Answers to our original research questions were produced by the results of the user requirements activities and by the preliminary evaluations. A summative presentation of the answers based on the participant sample in our study is provided below:

**R1: What are the habits of elderly people with regard to self-monitoring their health at home?**
  - Elderly people use common medical devices at home for the self-monitor of their health, such as blood pressure meter, blood glucose meter, and oximeter.
  - Most elderly people who self-monitor their health tend to share their monitoring results either with their doctor or with a person from their close environment and sometimes with both.
  - Elderly people do not necessarily keep a record of their measurements in a consistent manner.
  - For elderly people, quality of life is viewed as being able to live an independent life, being self-sufficient and able to do daily activities, being in good health, and be close to your family.
**R2: What would elderly users require from an mHealth solution?**
  - An mHealth system should be able to automatically notify the user of health measurement results. However, the system should be adaptive and flexible to accommodate personal preferences as to when it should notify users.
  - Elderly people are open to the idea of sharing their health data with people of their close environment (e.g., relative, caregiver, health care provider) and an mHealth system should provide such capability. However, they want to be in control of whom they share these data and under what conditions.
  - Aging is a continuous process and each person experiences it differently and has different needs over time. Elderly people require an mHealth solution that is flexible and expandable over time. For example, a supported health measurement that was not needed in the past may become important in a future time and vice versa. The solution should allow the elderly person to decide what is best for him/her at any given time by providing such options in the preferences.
  - Although, elderly people as we found out from our study are not averse to modern technologies and many are already using them on a daily basis, there is still a very wide range of skills observed in this user group. There are elderly people who have never used a touch screen before and elderly people who make skype calls to their grandchildren or do their banking from their smartphone. Regardless of the level of skills of the user, an mHealth system should strive for a good balance between simplicity, intuitiveness and available functionality. It should be able to show important data in an aggregate format, so that the user can get a feeling of his/her daily health status at a single glimpse, without becoming overwhelmed.
**R3: Are healthcare professionals and caregivers positive towards employing such mHealth solutions for self-monitoring of health status when it comes to their older patients?**
  - From our experience, both these groups are equally interesting and positive towards exploring mHealth solutions for health self-monitoring and remote monitoring purposes. They recognise the diverse needs of elderly people and at the same the shift in economics due to the aging population problem. So, they are positive towards applications that would support living independently and reducing hospital visits or hospital stays.
  - Health professionals are also positive towards the use of commercially available wearable devices as self-monitoring tools, recognising the advances in sensor technologies employed by them.
  - Health professionals recognise the need for elderly people to become proactive in self-managing their health and for easy to use tools that will allow them to do so.

We acknowledge that there are a couple of limitations to our study. Firstly, the study was conducted in Greece as such, the results do not reflect on the preferences, perceptions, or habits in regards to home health monitoring practices of older adults worldwide. Secondly, with regard to the usability evaluation, it should be noted that it was carried out using interactive prototypes without employing real health data from participants. The main objective was to assess the overall usability and acceptance of the proposed system. Future evaluations will address this limitation.

In the near future, the current version of the system’s components will undertake additional more extensive user-based evaluations to further improve the usability and user experience for all user groups. In the long run, it is of interest to us to evaluate the MHW system in the context of a long-term pilot that will focus on assessing its potential for the improvement of the quality of life of elders. Such a study would require the involvement of elderly people and their physicians and caregivers who would be given the system to try it in their environments and in real conditions for a long period of time.

## Figures and Tables

**Table 1:** Characteristics of elderly participants.

Gender		Living Situation		Age Range	
Male:	17	I live in a flat/home on my own:	8	65–69:	6
Female:	28	I live in a flat/home with a relative:	19	70–74:	12
		I live in a flat with a caregiver:		75–80:	6
		I live in a flat/home on my own, but receive help from a relative or caregiver:	1	80+:	21
		I live in a nursing home:	17		

**Table 2:** Type of technologies and electronic applications used by elderly participants.

Technology Usage					
	**Smartphone or Tablet**	PC	Emails	Social Media Apps	Other Mobile Apps
Daily:	11	8	3	4	7
1–2 times per week:	1	1	1	2	3
1–2 times per month:	-	-	3	2	-

**Table 3:** Types of medical devices used at home (select multiple options allowed).

Medical Devices	Frequency
Blood pressure meter	33
Blood sugar meter	16
Oximeter	4
Blood pressure and blood sugar meter	12

**Table 4:** Measurements recording methods employed at home.

Methods of Recording Results at Home	Frequency	Percentage
Manually	14	40%
Recording on the device	2	6%
I do not record	19	54%
Total	35	

**Table 5:** Sharing of home health measurements results with others (select multiple answers allowed).

Sharing of Results with	Frequency
My physician (only)	17
A relative (only)	5
My physician and a relative/caregiver	12
No-one	9

**Table 6:** System notifications for health measurements preferences.

System Notifications about Results	Frequency	Percentage
Daily (1–2 times)	17	39%
Only for abnormal results	17	39%
On demand	7	16%
Other	3	7%
Total	44	

**Table 7:** Preferences for sharing health data with others—hypothetical scenario (select multiple answers allowed).

Sharing of Results with	Frequency
My physician (only)	12
My physician and a relative	10
My physician and my home assistant or caregiver	4
A relative (only)	9
Home assistant or caregiver (only)	1
No-one	3

**Table 8:** Preferred method of notifying other people about health measurements results.

Notification Method	Frequency	Percentage
Automatically for all measurements	22	50%
Automatically but ONLY for abnormal results	11	25%
Automatically for selected measurements	1	0,4%
I want to choose when and to whom to send a notification	10	23%
Other	0	0%
Total	44	

**Table 9:** Characteristics of healthcare professionals.

**Gender**		**Specialty**		**Age Range**	
Male:	5	Occupational therapists:	2	25–34:	1
Female:	11	Physicians:	2	35–44:	8
Practice context		Nurses:	8	45–54:	5
Private sector:	8	Other: physical therapist:	1	55–64:	2
Public sector:	8	Other: social worker:	3	65+:	0

**Table 10:** Results of selected health measurements for monitoring (select multiple options allowed).

Health Measurements	Frequency
Blood pressure	15
Heart rate	15
Blood sugar	15
Blood Oxygen	13
Body temperature	10
Stress level (electro-dermal activity and dermal conductivity)	7
Detection of coughing	7
Other	0

**Table 11:** Additional measurements that would be of interest to the healthcare professionals (select multiple options allowed).

Other Measurements	Frequency
Water intake (e.g., glasses of water)	11
Physical activity (e.g., No of steps)	7
Weight	11
Number of cigarettes (if applicable)	9
Sleep	8
Falls	10
Other. Describe:	0

**Table 12:** Characteristics of participants from caregivers user group.

Gender		Age	Relation with Elderly		Frequency of Provided Care		
Male:	6	25–34:	4	Relative:	7	Daily:	6
Female:	4	35–44:	0	Home assistant:	1	1–2 times per week:	3
		45–54:	3	Caregiver:	1	1–2 times per month:	0
		55–64:	1	Other:	1	When needed:	1
		65+:	1				

**Table 13:** Type of assistance provided to cared ones.

Type of Assistance Provided	Frequency
Buying medicine and/or helping with medicine intake	7
Arranging visits to physicians	3
Accompanying elderly to physicians’ appointments	3
Monitoring of health measurements (e.g., taking bl. Pressure, bl. Sugar)	5
Other	1

**Table 14:** Health measurements for monitoring (select multiple options allowed).

Health Measurements	Frequency
Blood pressure	10
Heart rate	6
Blood sugar	3
Blood Oxygen	6
Body temperature	4
Stress level (electro-dermal activity and dermal conductivity)	7
Cough detection	2
Other	2

**Table 15:** Speech recognition performance.

Gender	Lavalier Microphone	Smartphone Microphone
Male subjects (4)	91.7% (88/96)	90.06% (87/96)
Female subjects (12)	94.1% (271/288)	93.75% (270/288)
Total	93.5% (359/384)	93.0% (357/384)

## Data Availability

The data are not available due to privacy and ethical restrictions.
